# Organomediated polymerization

**DOI:** 10.1038/s42004-024-01134-1

**Published:** 2024-03-21

**Authors:** Satoshi Honda, Karin Odelius, Haritz Sardon

**Affiliations:** 1 Graduate School of Arts and Sciences, The University of Tokyo, 3-8-1 Komaba, Meguro-ku, Tokyo 153-8902 Japan; 2 Department of Fibre and Polymer Technology, KTH Royal Institute of Technology, Teknikringen 56-58, 100 44, Stockholm, Sweden; 3 POLYMAT, University of the Basque Country UPV/EHU, Joxe Mari Korta Center, Avda. Tolosa 72, 20018 Donostia-San Sebastián, Spain; 4 Department of Polymers and Advanced Materials: Physics, Chemistry and Technology, Faculty of Chemistry, University of the Basque Country. UPV/EHU, Donostia-San Sebastián, 20018 Spain

**Keywords:** Polymer synthesis, Polymerization mechanisms, Organocatalysis

## Abstract

*Communications Chemistry* is pleased to introduce a Collection of articles focused on organomediated polymerization. Here, the Guest Editors highlight the themes within and look towards the future of this research field.

More than 100 years have passed since the concept of polymers was suggested by Staudinger^1^. Polymers are used worldwide because they are inexpensive, durable, lightweight, flexible, and easy to process. They have contributed to the advancement of human life and industry. Some predict that the global production of polymers will reach 800 million tons by 2040^2,3^. However, in the 1990s, environmental concerns began to increase, and as the amount of polymers used increased, the development of recyclable alternatives and environmentally friendly synthetic methodologies became recognized as critical issues. The challenge of synthesizing more sustainable polymers has been extended to both academia and industry and requires a wide range of approaches. One challenge in synthesizing more sustainable polymers is tied to the development of benign and efficient catalysts. Although transition metal-based catalysts have traditionally been used in polymer chemistry, their toxicity and, in some cases, limited availability, have created an urgent need to develop more efficient, abundant, and sustainable catalyst families. In this context, organocatalysis, or the use of organic molecules to mediate polymer reactions, has evolved as an effective complement to transition metal-based catalysis in polymerization, polymer functionalization, and depolymerization.

In this Collection, we are delighted to showcase a variety of cutting-edge studies that highlight important and exciting advances and challenges in this field. These contributions cover three main themes: polymerization, functionalization, and depolymerization.

## Polymerization

Since the seminal works of ref. 4 as well as ref. 5 showed that simple small organic molecules can catalyze ring-opening polymerization (ROP) of cyclic monomers^6^, the field has continued to explore efficient catalytic systems and expand available monomers to elicit novel functionalities. Additionally, precise control of their primary structures enables the production of polymers with uniform properties, contributing to the provision of consistent quality regardless of the production lot. In this regard, a study from Rheinberger et al. successfully performed ROP of phosphorus-containing monomers to achieve the fabrication of MRI-traceable nanomaterials^7^. In addition, the novel concept of quasi-alternating copolymerization of oxiranes reported by Fornaciari et al. provides important insights into the control of the primary structure of polymers through the effective use of organocatalysts^8^. On the research aspect of pursuing a more efficient catalyst system, a new mechanism of ROP based on an activation—deactivation equilibrium between dormant and propagating species, as seen in living radical polymerization, is reported by Okamoto et al. ^9^ As seen in a Review article by ref. 10, there are few examples of organocatalytic enantioselective ROP, and further development is desirable in the future. We also expect to see more and more practical-oriented research, such as the application of ROP to sustainable vat-photopolymerization 3D printing systems^11^.

## Functionalization

Organocatalysts are also important for effectively transforming existing raw materials into useful monomers or polymers. In this Collection, Scheelje and Meier report the formation of five-membered cyclic carbonates from carbon dioxide and naturally abundant limonene, followed by the synthesis of urethane-containing monomers by organocatalytic ring-opening reactions, and then polyurethanes by polycondensations^12^. The conversion of abundant natural resources into monomers and polymers and the chemical fixation of carbon dioxide are themes that have been opened up by the use of metal-based catalysts and will continue to be important in the future with the development of efficient organocatalysts. The synthesis of vitrimers via organocatalytic depolymerization of polyethylene terephthalate (PET) and subsequent transesterification using an earth-abundant metal-based catalyst reported by Ng et al. highlights the importance of combining functionalization and depolymerization concepts in upcycling^13^.

## Depolymerization

The above two topics focused on how to efficiently synthesize polymers from monomers or how to functionalize the resulting polymers by using organocatalysts. These could be characterized as the process of chemical transformation of raw materials or precursor polymers into useful polymeric materials. In contrast, the topic of depolymerization focuses on how to efficiently return the finished polymer products to raw materials or precursors. These aspects are directly related to the concept of chemical recycling, for which effective organocatalytic depolymerization and related chemical decomposition reactions are key. In this Collection, relevant state-of-the-art original articles include the organocatalytic chemical decomposition of superengineering plastics by refs. 14,15 and the enzymatic degradation of PET by ref. 16. On the other hand, the depolymerizability of polymers sometimes impairs their performance to meet industry requirements. As highlighted by ref. 17, recent progress in the synthesis of chemically recyclable polyacetals enabled the production of ultra-high molecular weight poly(1,3-dioxolane) with industrially relevant physical properties^18^. Further work in the relevant research direction will seek to expand the scope of chemically recyclable polymers with industrially relevant properties.

## Outlook

Through various studies in recent years, we can witness that research on organocatalysts has made significant achievements not only in polymerization reactions, but also in the functionalization and depolymerization, and has contributed to the recycling and upcycling of polymeric materials. This Collection aims to reaffirm the usefulness of organocatalysts from these three directions. It is anticipated that the realization of a sustainable society will require further refinement and integration of all these directions. Although it is still in its infancy, the fact that studies on upcycling that combine depolymerization and functionalization are reported in this Collection is evidence that such integrated research is steadily emerging. We expect the emergence of sophisticated systems that efficiently manipulate polymerization, functionalization of raw materials and polymers, and even depolymerization, all by utilizing organocatalysts in the future.
